# Labor and childbirth care for women deprived of liberty: a scoping review

**DOI:** 10.1590/1980-220X-REEUSP-2024-0035en

**Published:** 2024-09-13

**Authors:** Tatyanne Maria Pereira de Oliveira, Joel Araújo dos Santos, Priscila de Souza Aquino, Herla Maria Furtado Jorge

**Affiliations:** 1 Universidade Federal do Piauí, Departamento de Enfermagem, Teresina, PI, Brazil.; 2 Universidade Federal do Ceará, Departamento de Enfermagem, Fortaleza, CE, Brazil.

**Keywords:** Pregnant Women, Pregnant Women, Labor, Obstetric, Parturition, Prisons, Mujeres Embarazadas, Mujeres Embarazadas, Trabajo de Parto, Parto, Prisiones

## Abstract

**Objective:**

To map and analyze scientific evidence on care provided to women deprived of liberty during labor and childbirth.

**Method:**

A scoping review, developed in accordance with JBI methodology, whose information sources were accessed in databases and gray literature. Selection was carried out between October and December 2023, based on reading titles, abstracts and descriptors, considering the following eligibility criteria: articles, dissertations and theses with different methodological designs available in full, without language and time limitations. Analysis was conducted by two independent reviewers, using inductive content analysis.

**Results:**

Fifteen studies were included. From the synthesis of results, two categories emerged: From the cell to the delivery room: care for women deprived of liberty; Experiences of women deprived of liberty during labor and childbirth.

**Conclusion:**

This study highlights the fragility of care practices during labor and childbirth, imposing significant challenges and resulting in adverse experiences that compromise the quality of motherhood and violate women’s fundamental rights.

## RESUMEN

**Objetivo:**

Mapear y analizar evidencia científica sobre la asistencia brindada a mujeres privadas de libertad durante el parto y el nacimiento.

**Método:**

Revisión de alcance, desarrollada de acuerdo con el método JBI, a cuyas fuentes de información se accedió en bases de datos y literatura gris. La selección se realizó entre octubre y diciembre de 2023, a partir de la lectura de títulos, resúmenes y descriptores, considerando los siguientes criterios de elegibilidad: artículos, disertaciones y tesis con diferentes diseños metodológicos disponibles en su totalidad, sin límite de idioma ni de tiempo. El análisis fue realizado por dos revisores independientes, utilizando análisis de contenido inductivo.

**Resultados:**

Se incluyeron 15 estudios. De la síntesis de resultados surgieron dos categorías: De la celda a la sala de partos: asistencia a mujeres privadas de libertad; Experiencias de mujeres privadas de libertad durante el trabajo de parto y el parto.

**Conclusión:**

Este estudio resalta la fragilidad de las prácticas de cuidado durante el trabajo de parto y el nacimiento, imponiendo importantes desafíos y resultando en experiencias adversas que comprometen la calidad de la maternidad y violan los derechos fundamentales de las mujeres.

## INTRODUCTION

Ensuring access to healthcare is a fundamental right that must be guaranteed to all people^(1)^. However, for specific groups of the population, such as women deprived of liberty, there are obstacles that result in poor healthcare^(2)^. Female incarceration intensifies challenges related to social inequalities and gender-based violence, especially with regard to sexual and reproductive rights^(3)^.

In the international scenario, despite public policies that ensure the rights of women deprived of liberty, exemplified by the Mandela Rules of 1995 and the Bangkok Rules, enacted in 2010, both established by the United Nations (UN)^(4,5)^, the implementation of these guidelines in prisons continues to face several challenges. These challenges include vulnerabilities in women’s healthcare during pregnancy and the postpartum period, the occurrence of obstetric violence, experiences of abandonment at the time of childbirth and inadequacies in prison settings^(6)^.

The literature indicates an increase in the number of pregnant women in prison, with a global growth rate that rose from 7.2% in 2006 to 8.8% in 2014^(7)^. Prison settings are characterized by unfavorable conditions, marked by inadequate and unsanitary spaces. These conditions not only represent a challenge to pregnant women’s health, but also have the potential to trigger significant biopsychosocial changes^(8)^, being associated with the development of maternal-fetal complications^(9)^.

In the prison context, pregnant women are exposed to verbal, physical and psychological violence during prenatal care, which has significant implications during labor and childbirth. Practices such as physical restraint stand out, followed by neglect of necessary care during the postpartum period^(7)^. These practices are not aligned with the principles of safe childbirth^(10,11)^.

The challenges faced by pregnant women in prisons, marked by limited access to healthcare and the presence of psychological and moral violence, highlight the pressing need to address this gap. These challenges not only impact the social and health conditions of women deprived of liberty, but also have repercussions on the lives of their children^(12)^.

In this context, the importance of providing care to women in prison during labor becomes evident, especially due to the lack of specific information. It is worth noting the lack of ongoing or completed scoping studies on this topic. Thus, this study aimed to map and analyze scientific evidence on the care provided to women deprived of liberty during labor and childbirth.

## METHOD

### Study Design

This is a scoping review, prepared in accordance with JBI recommendations, which aims to map scientific evidence, main concepts and gaps on a given topic^(13)^. Aiming at study quality and transparency, the Preferred Reporting Items for Systematic Reviews and Meta-Analyses extension for Scoping Reviews (PRISMA-ScR) checklist guidelines were used^(14)^. The research protocol was registered in the Open Science Framework (OSF), with DOI identification: https://doi.org/10.17605/OSF.IO/MKAD5.

It is important to note that the scoping review represents a process of knowledge synthesis. Its main purpose is not to critically assess the quality of this evidence, but rather to comprehensively understand the set of evidence found^(14,15)^. To this end, it was carried out following five stages: 1) research question elaboration; 2) relevant study identification; 3) study selection; 4) information organization; 5) synthesis and presentation of results^(16)^.

### Research Question Identification

The research question was developed based on the mnemonic strategy PCC, which corresponds to: P (Population): pregnant women or women in labor; C (Concept): care during labor and childbirth; and C (Context): deprivation of liberty. Thus, the following question was listed: what does the scientific literature portray about care for women deprived of liberty during labor and childbirth?

### Selection Criteria

Articles, dissertations and theses with different methodological designs related to labor and childbirth care for women deprived of liberty, available in full, without language or time restrictions, were included. Abstracts of conference proceedings, editorials, response letters, theoretical reflections, course completion papers and those that did not answer the research question were excluded.

### Data Collection and Study Selection

To carry out the searches, controlled words were used from the Health Science Descriptors (DeCS), Medical Subject Headings (MeSH), List of Headings from CINAHL Information Systems, Embase Subject Headings (EMTREE), identifying the controlled descriptors and their keywords.

Searches were conducted between October and December 2023 in the following databases: Medical Literature Analysis and Retrieval System Online (MEDLINE) via National Center for Biotechnology Information (NCBI/PubMed); Web of Science (WoS); Cumulative Index to Nursing and Allied Health Literature (CINAHL); and Excerpta Medica dataBASE (EMBASE). The journals were accessed through the Coordination for the Improvement of Higher Education Personnel (CAPES – *Coordenação de Aperfeiçoamento de Pessoal de Nível Superior*) Journal Portal, *Literatura Latino-Americana e do Caribe em Ciências da Saúde* (LILACS), *Base de Dados em Enfermagem* (BDENF), *Índice Bibliográfico Español en Ciencias de la Salud* (IBECS) via the Virtual Health Library (VHL). Gray literature was also a source of searches through *Biblioteca Digital de Teses e Dissertações* (BDTD) and Google Scholar.

The databases were searched using controlled descriptors and their keywords, combined with the Boolean operators “OR” and “AND” to compose the search strategy. Chart 1 presents the high-sensitivity search expression performed in MEDLINE/PubMed, which was adapted for the other selected databases according to their specificities and can be verified in the scoping review protocol: https://doi.org/10.17605/OSF.IO/MKAD5.

**Chart 1:** High sensitivity search expression performed in MEDLINE/PubMed – Teresina, PI, Brazil, 2023.

MEDLINE/PubMed
((“Pregnant Women”[Mesh] OR (Pregnant Woman) OR (Woman, Pregnant) OR (Women, Pregnant) OR “Pregnancy”[Mesh] OR (Pregnancies) OR (Gestation)) AND (“Labor, Obstetric”[Mesh] OR (Obstetric Labor) OR “Delivery, Obstetric”[Mesh] OR (Deliveries, Obstetric) OR (Obstetric Deliveries) OR (Obstetric Delivery) OR “Parturition”[Mesh] OR (Parturitions) OR (Birth) OR (Births) OR (Childbirth) OR (Childbirths))) AND (“Prisons”[Mesh] OR (Prison) OR (Penitentiaries) OR (Penitentiary) OR “Prisoners”[Mesh] OR (Prisoner) OR (Hostages) OR (Hostage) OR “Correctional Facilities”[Mesh] OR (Correctional Facility) OR (Facilities, Correctional) OR (Facility, Correctional) OR (Penal Institutions) OR (Institution, Penal) OR (Institutions, Penal) OR (Penal Institution) OR (Correctional Institutions) OR (Correctional Institution) OR (Institution, Correctional) OR (Institutions, Correctional))

After searching the databases, the results were imported into a reference management program, EndNote^®^ Web, to identify duplicates. The results were then imported into Rayyan^®^ of the Qatar Computing Research Institute (QCRI)^(17)^ for study analysis, selection and exclusion. It is important to note that the stages were conducted by two independent reviewers. Cases of disagreement were resolved with the help of a third reviewer, before proceeding to the full reading and inclusion of the studies in the review.

The first selection was made by reading titles and abstracts to analyze the authors’ agreement. Cohen’s Kappa coefficient was calculated^(18)^, which presented a value of 0.85 (strong agreement). Subsequently, the selected articles were read in full and assessed according to inclusion and exclusion criteria.

The data were extracted, according to the instrument adapted from the JBI manual^(13)^, in synoptic tables in Microsoft Excel^®^ containing information on authorship, journal, country of origin, year of publication, study title, objective, research design, sample number and care regarding labor and childbirth.

### Data Analysis

The data were subjected to inductive content analysis^(19)^. This process was structured according to the three proposed stages: data preparation; organization; and report. In the initial preparation stage, the data were organized into synoptic tables, according to the previously established information. Then, during the organization stage, the main results were identified and submitted to open coding for subsequent categorization. Finally, in the report preparation stage, which corresponds to the final writing of the material, the results were presented descriptively through charts and texts, and were grouped into common thematic categories, providing a clear and concise summary of the findings. Moreover, we used the *Interface de R pour les Analyses Multidimensionnelles de Textes et de Questionnaires* (IRaMuTeQ)^(20)^ only to create a word cloud based on the results obtained on labor and childbirth care from selected studies.

As this is a scoping review study, there was no need for assessment by a Research Ethics Committee (REC).

## RESULTS

**Figure 1:**
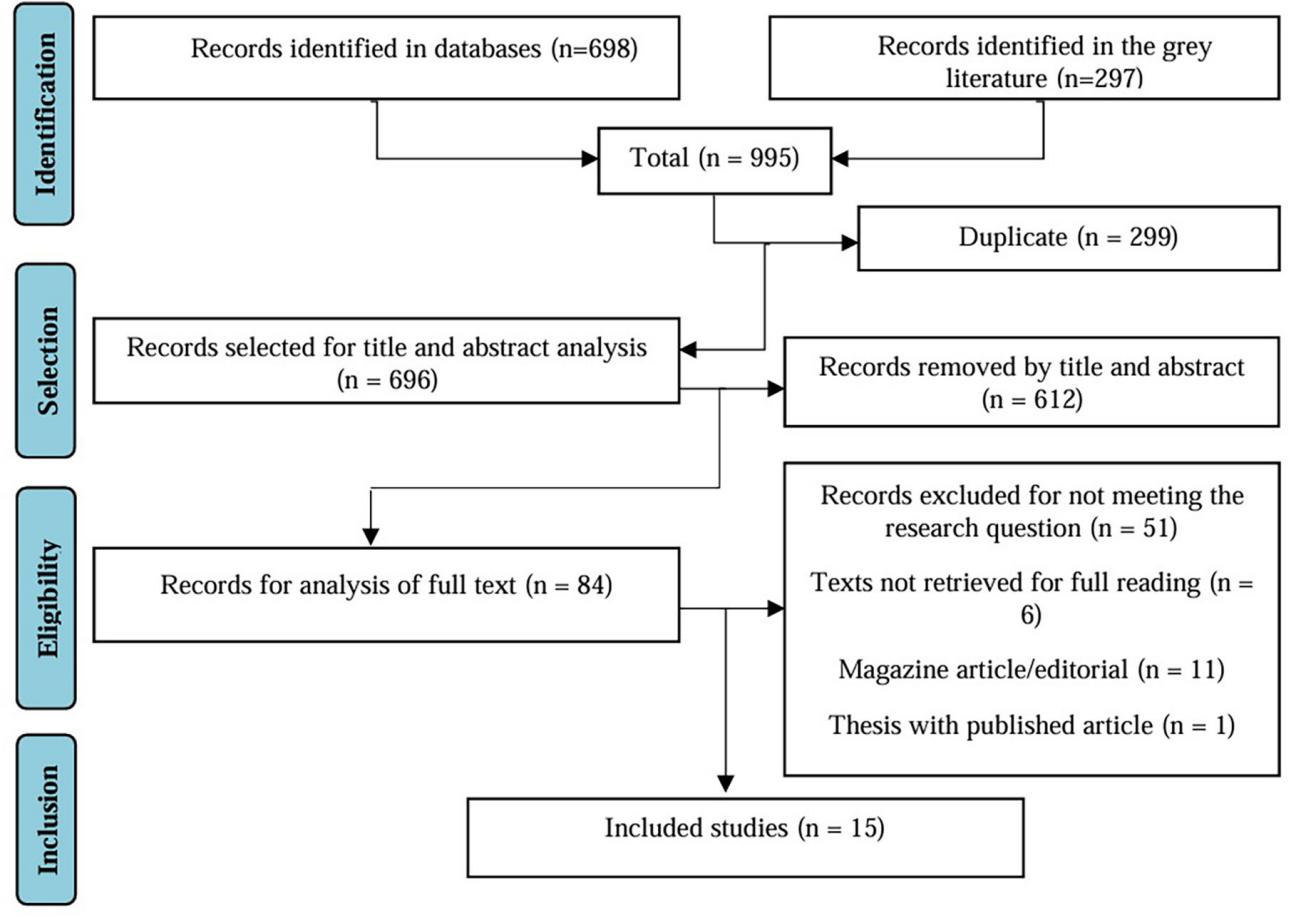
Flowchart of the study selection process according to the Preferred Reporting Items for Systematic Reviews and Meta-Analyses extension for Scoping Reviews (PRISMA-ScR) – Teresina, PI, Brazil, 2023.

A total of 995 studies were identified from the search strategy, of which 195 were in MEDLINE/PubMed, 135 in WoS, 150 in CINAHL, 123 in EMBASE, 51 in LILACS, 32 in BDENF, 12 in IBECS, 102 in BDTD and 195 in Google Scholar. A total of 299 records were excluded due to duplication. A total of 696 studies were eligible for the title and abstract analysis stage; of these, 612 studies were excluded. Therefore, 84 were selected for full-text reading and analysis for inclusion in the review, of which 69 were excluded. Thus, 15 studies were selected for the final synthesis. The selection stages were performed according to the PRISMA-ScR flowchart, as described in Figure 1.

Chart 2 shows the 15 studies that made up the final research sample. From the analysis of these studies, the following categories emerged: “From the cell to the delivery room: care for women deprived of liberty”^(21–24,27,29,30,33)^; and “Experiences of women deprived of liberty during labor and childbirth”^(21–28,30–35)^.

**Chart 2:** Characterization of research studies that make up the study sample according to authorship data, journal, country and year, article title, objectives, research design, sample number and labor and childbirth care – Teresina, PI, Brazil, 2023 (n = 15).

Authorship/journal/country and year	Title	Objective	Research design/sample number	Care with labor and childbirth
Abbott et al.^(21)^. Birth, United Kingdom/2023	Experiences of midwifery care in English prisons	Examine the experiences and perceptions of obstetric care for pregnant women in detention and custodial staff in English prisons.	Qualitative study (ethnography)/28 women	– No choice of partner was given for childbirth and birth; – There was no choice of obstetric care provider or place of birth; – Both women and staff demonstrated a lack of awareness of the rights a pregnant woman should receive.
Kramer et al.^(22)^. Matern Child Health J. United States/2023	Shackling and pregnancy care policies in US prisons and jails	Assess pregnancy policies and practices in prisons and jails with an emphasis on the use of restraints and compliance with anti-shackling legislation.	Qualitative study/28 units	– Restraints were used during pregnancy and post-partum, including during transport; – Significant isolation for most women giving birth in custody; – Did not require that the officers present during the childbirth be female.
Cavanagh et al.^(23)^. Soc Sci Med. Canada/2022	Lived experiences of pregnancy and prison through a reproductive justice lens: A qualitative meta-synthesis	Better understand how incarcerated people experience pregnancy and how their experiences are represented in research.	Systematic review (metasynthesis)/31 articles	– Labor and birth were traumatic for many women, exacerbated by the intersection of prison policies and clinical practices that disregarded their bodily autonomy and their role as mothers; – One participant described being scheduled and transported to the hospital to give birth without knowing what would happen.
Dalenogare et al.^(24)^. *Ciên Saúde Colet*. Brazil/2022	*Pertencimentos sociais e vulnerabilidades em experie^ncias de parto e gestac¸a~o na prisa~o*	Understand the pregnancy and childbirth experiences of women in prison.	Qualitative study/seven women	– Labor was perceived as painful and distressing, due to the degrading state of the care offered from the removal from the prison unit to the health institution; – In the penitentiary, labor progresses with the help of professionals from the Prison Basic Health Unit; – During the night or on weekends, the decision to refer women to the health institution is made by security agents; – Absence of companions of women’s choosing and, often, without being able to inform them about the situation, they are accompanied by security agents; – Use of shackles on the way from the prison to the health institution; – The treatment offered by the health institution staff is perceived in different ways by women. Some praised it, while others reported being treated with indifference, neglect and violence; – Inattention to good practices related to childbirth, with the use of interventionist measures to speed it up.
Kirubarajan et al.^(25)^. BJOG-Int J Obstet Gy. Canada/2022	Pregnancy and childbirth during incarceration: A qualitative systematic review of lived experiences	Characterize patients’ experiences of pregnancy and childbirth during incarceration through qualitative synthesis.	Systematic review (metasynthesis)/24 articles	– Use of restraints during pregnancy and childbirth; – Lack of emotional support; – Trauma of separation from the newborn after birth.
Fortunato et al.^(26)^. REAS. Brazil/2022	*Percepção das mulheres privadas de liberdade sobre a assistência à saúde recebida no pré-natal, parto e puerpério: revisão integrativa*	Describe, from the perspective of women deprived of liberty, how healthcare occurs during pregnancy, childbirth and the puerperium.	Integrative review/15 articles	– Delay in referral to maternity; – Family members are not informed of the start of labor and, when notified, they are unable to arrive in time to accompany women; – The police escort does not make contact with the family, and these women rarely receive visits while still in the maternity ward; – Women highlighted violence, mainly in verbal and psychological forms, in addition to invasive procedures, physical aggression and negligence, lack of humanization and guidance during care; – There are reports of the use of shackles during childbirth and hospitalization.
Suarez^(27)^. Correct Health Care. United States/2021	“I Wish I Could Hold Your Hand”: Inconsistent Interactions Between Pregnant Women and Prison Officers	Explore women’s interactions with prison officers during pregnancy, labor and childbirth.	Qualitative study/18 women	– Rude officers in the unit during transports and even in the delivery room; – General lack of privacy and acknowledgement that they were giving birth; – Police officers were sometimes nosy or too talkative or ignored women in labor who were often in pain.
Abbott et al.^(28)^. Sociol Health Illn. United Kingdom/2020	Pregnancy and childbirth in English prisons: institutional ignominy and the pains of imprisonment	Explore the experiences of pregnant women in prison through qualitative interviews with a sample of female detainees, another sample of prison staff, and field observations.	Qualitative study (ethnography)/28 women	– Loss of privacy compounded the loss of dignity and decency; – The environment was considered so hostile to spontaneous labor that it seemed unsafe to engage in labor in prison.
Johnston^(29)^. Criminol Crim Justice. United Kingdom/2019	Imprisoned mothers in Victorian England, 1853–1900: Motherhood, identity and the convict prison	Explore the experiences of mothers incarcerated in the Victorian convict prison system.	Qualitative study (life story)/288 women	– Pregnant women when arrested or convicted gave birth in local prisons.
Matos et al.^(30)^. Interface. Brazil/2019	*Filhos do cárcere: representac¸o~es sociais de mulheres sobre parir na prisa~o*	Understand the social representations of incarcerated pregnant and postpartum women about giving birth in prison.	Qualitative study/19 women	– Only prison officers are present when the time comes to give birth and they are taken to the hospital; – Perceive the judgment that was made, denoting the prejudice of other postpartum women, companions and healthcare professionals towards them.
Leal et al.^(31)^. *Ciên Saúde Colet*. Brazil/2016	*Nascimento na prisão: gravidez e nascimento atrás das grades no Brasil*	Outline the profile of the female incarcerated population living with their children in women’s prisons in the capitals and metropolitan regions of Brazil as well as the conditions and practices related to care during pregnancy and childbirth during incarceration.	Quantitative study/241 mothers	– The presence of companions of women’s choice during hospitalization for childbirth was 3%; – Postpartum women reported having suffered mistreatment or violence during their stay in maternity wards by healthcare professionals (16%) and by guards or prison officers (14%); – The use of shackles at some point during hospitalization for childbirth was reported by 36% of pregnant women, with 8% reporting having been put shackles even during childbirth; – Only 10% and 11% of women reported having their privacy respected by healthcare professionals and prison guards/officers, respectively. This percentage was slightly higher when the topic was healthcare professionals’ treatment towards them (18%).
Spinola^(32)^. *Faculdade de Medicina da USP*. Brazil/2016	*A experiência da maternidade no cárcere: cotidiano e trajetórias de vida*	Know and understand the experience of motherhood in prison based on the daily lives and life trajectories of women released from the penitentiary system.	Qualitative study (hermeneutics)/two women	– Compliance with all bureaucratic procedures (signatures, magazines) and then being sent to the hospital; – The condition of being taken to the hospital only occurred when a child was about to be born; – Use of hand and foot cuffs during labor and childbirth; – Feeling of pain and no proper communication; – Description of difficult childbirth.
Ferszt and Clarke^(33)^. J Health Care Poor Underserved. United States/2012	Health care of pregnant women in U.S. state prisons	Examine healthcare practices for pregnant women in state prisons.	Mixed-methods study/32 prisons	– Use of abdominal chains/belts, leg shackles, and shackles when transporting women to a hospital or clinic; – Restraint during labor and even during the birth of a baby; – Restraint during immediate recovery period and in hospital rooms.
Rosinski et al.^(34)^ *Ciênc., Cuid. Saúde*. Brazil/2006	*Nascimento atra´s das grades: uma pra´tica de cuidado direcionada a gestantes, pue´rperas e rece´m-nascidos em privac¸a~o de liberdade*	Develop a care practice aimed at pregnant women, postpartum women and newborns deprived of liberty, guided by Orem’s General Nursing Theory.	Qualitative study/12 women	– Use of shackles during labor and childbirth that made it impossible to hold the baby; – Lack of contact with a child.
Amnesty International^(35)^. Birth. United States/2000	Pregnant and imprisoned in the United States	Describe human rights violations of pregnant women and mothers incarcerated in prisons and jails in the United States.	Qualitative study/-	– Pregnant women were restrained when transported to the hospital and kept under restraints while in the hospital, even while at labor, unless a doctor ordered their removal and a correctional officer approved; – Women were put shackles even in the presence of a prison guard or, in some cases, shackles were removed up to 30 minutes before childbirth; – Lack of permission to move around during labor; – Some women reported that, after birth, they remained with the baby for a while, but, shortly afterwards, the police replaced the shackles to remove the baby from the delivery room.

As for study design, 14 articles and one dissertation were selected. The year of publication ranged from 2000 to 2023, with a predominance of studies in 2022 (n = 4). Regarding the place of publication, Brazil was the country with the largest number of studies (n = 6), followed by the United States (n = 4), the United Kingdom (n = 3) and Canada (n = 2). In relation to language, most studies were published in English (n = 9) and Portuguese (n = 6). Regarding the methodological characteristics of studies included in this study, qualitative research (n = 10), reviews (n = 3), mixed-methods studies (n = 1) and quantitative studies (n = 1) stood out.

### From the Cell to the Childbirth Room: Care to Women Deprived of Liberty

Childbirth represents a sudden rupture of the bond established prenatally, and is traumatic for many women due to prison policies and clinical practices that disregard their bodily autonomy and their role as mothers^(23)^. Care with labor and childbirth begins in prison settings, where healthcare professionals from the prison unit monitor labor and childbirth, advising the appropriate time to refer patients to reference hospitals. However, at night or on weekends, this decision is made by security agents^(24,30)^.

In this context, care is characterized by a lack of privacy, disrespect for labor and negligence or disregard for reported pain, contributing to delays in care, increasing the feeling of abandonment and, consequently, leading some women to give birth alone, without care^(27)^, and often pregnant women give birth in the prison itself^(29)^.

Currently, it is recommended that women in labor be transferred to hospital care. However, it is observed that transportation is often carried out inappropriately, including practices such as restricting movement with the use of restraints (shackles, chains, handcuffs) on the legs and the use of abdominal chains/belts, despite international understanding and legal prohibitions to the contrary^(22,24,33)^.

The inclusion of a male security agent during transport was mentioned as a reduction in autonomy and a lack of choice, causing discomfort, insecurity and anxiety, constituting a source of stress^(21)^. Childbirth is recognized as a time of anguish, pain and loneliness, not only due to physiological aspects, but also due to degradation in the quality of care offered, despite some reports of effective care^(24)^.

### Experiences of Women Deprived of Liberty During Labor and Childbirth

Women witness the neglect of good practices related to childbirth, which is aggravated by the use of restraints, such as shackles and chains, in bed during the birthing process. Reports mention that legs, hands and even the spine (abdomen) are places of chaining^(22,24–26,31,32,34,35)^. It is important to note that containers are only removed upon medical request^(22,33,35)^.

Another issue highlighted is the absence of a freely chosen companion or the presence of a male security agent, which indicates significant isolation, which can result in traumatic, humiliating experiences and trigger sexual trauma^(22,26–28)^. Furthermore, early contact between mother and baby is minimal or non-existent^(25,34)^, which can have negative impacts on the child’s development and also on women. Furthermore, after giving birth, some women were put shackles again, with separation from the baby described as a traumatic and devastating event for mothers^(25)^.

Both women and others involved demonstrate a lack of awareness regarding the rights that should be guaranteed to a pregnant woman^(21)^. These aspects contribute to women recognizing their lack of preparation during childbirth, with a general lack of knowledge of the process^(30)^.

**Figure 2:**
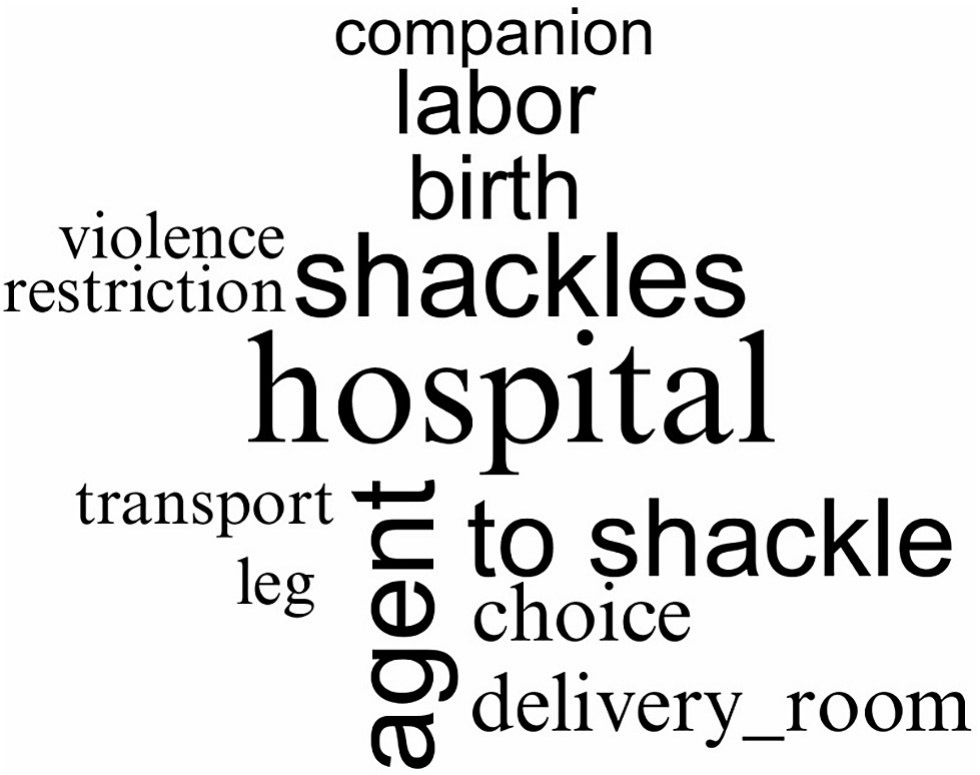
Word cloud of studies on care during labor and childbirth – Teresina, PI, Brazil, 2023.

Figure 2 illustrates the main words identified in study analysis about care during labor and childbirth for women deprived of liberty.

## DISCUSSION

Caring for women deprived of liberty is marked by significant challenges, as studies point out^(21–24)^. The situation of deprivation is often characterized by severe restrictions, social isolation and consequences for legal transgressions. Implicitly, the image of deprivation of rights, mistreatment and pain emerges. This perception extends to care for pregnant women deprived of liberty, from prenatal to post-partum.

For a comprehensive analysis of the situation, it is crucial to examine the health of women in prison settings in general. A study conducted in Ceará, Brazil, in 2018^(2)^, with women deprived of liberty revealed that the quality of healthcare in the prison facilities investigated is unsatisfactory. In addition to neglecting the women’s specific needs, there is a significant lack of basic care, such as adequate food, clothing, hygiene products and medications.

The female prison population requires a specific approach that takes into account their social and cultural particularities, as these are crucial factors in planning appropriate interventions^(2)^. By focusing only on the dimension of motherhood as part of female identity, prison institutions reinforce gender stereotypes present in society in general, limiting the vision of women only to the role of mothers^(36)^.

In relation to childbirth, considered a period of anguish, pain and loneliness, it represents a sudden rupture of the bond established during prenatal care. It is often described as a traumatic experience^(23)^ for many incarcerated women, not only due to physiological factors, but also due to the lack of quality in the care provided^(37)^.

The literature highlights the precarious social conditions of mothers who gave birth in the context of deprivation of liberty. Among several issues, limited access to healthcare, the use of shackles during labor and childbirth, together with reports of violence and negative assessments of care, indicates that healthcare services have not effectively played their role as a protective barrier and guarantor of the rights of this segment of the population^(6,38,39)^.

Studies conducted in Peru^(40)^, the United Kingdom^(41)^ and the United States^(42)^ indicate that, after a pregnancy diagnosis, women deprived of liberty are transferred to maternity units that are more suited to their needs; however, not all of them have guaranteed access to places in these units. In the Brazilian context, in most states, pregnant women are also transferred to prison units specifically for mothers with children^(6,9)^. Regarding the time of childbirth, these women are sent to public hospitals^(37,43,44,45)^ and, after birth, they return to the same prison unit, where they often remain with their children. Afterwards, children are usually handed over to their maternal or paternal relatives and, in their absence, sent to shelters, whereas mothers return to the prison of origin^(31)^.

Alongside international normative instruments, such as the Bangkok Rules^(5)^, and national policies on reproductive rights in prisons, which, although important, are little respected in Brazil, the Interministerial Ordinance of January 16, 2014^(46)^ represents a significant change in the approach to issues related to female incarceration in the country^(31)^, since continuous increase in the number of women deprived of liberty and the recognition of the complexity of this phenomenon highlight the urgency of a broader reflection on issues related to pregnancy and motherhood in this context^(36)^.

In the analysis of the second category, related to experiences of women deprived of freedom during labor and childbirth, it is clear that they often describe their maternal experiences as a sequence of challenges faced throughout the entire gestational period^(23,31)^. The results reveal a series of difficulties that not only compromise the quality of motherhood, but also violate the fundamental rights of these women, manifesting themselves through physical, verbal and psychological violence.

Among the main forms of violence, the practice of imposing physical restraints is worrying, as it goes beyond the limits of safety, compromising the body physiological adjustment due to limited movement, intensifying pain and hindering the childbirth dynamics. Considering the cultural, legal and institutional diversity between different countries, in the Brazilian context where the largest number of studies were concentrated, Law 13.434/2017 prohibits the use of shackles during labor, childbirth and immediate postpartum period^(47)^. In Canada and the United Kingdom, jurisdictional guidelines address the use of shackles and/or physical restraints, advocating their non-use, but allowing their use when deemed necessary^(48,49 50)^. In the United States, the law is not uniform across states, resulting in a variety of laws that mention prohibition and use at some point during pregnancy and childbirth^(48)^.

Hence, although the UN prohibits the use of shackles during labor and childbirth^(5)^, the legal diversity between countries contributes to disparities in provision of healthcare to pregnant women and women in labor who are deprived of their liberty.

Moreover, women in prison have reported other forms of obstetric violence, such as the lack of a companion of choice during childbirth^(20,23,25,30)^. In contrast, in the UK, some prisons take a different approach, allowing women to request the presence of a trusted prison officer during labor^(51)^. In special situations, such as Maternal-Baby Units, when women are able to join, they have access to childbirth companions linked to charitable institutions, providing essential support during this very significant moment^(41)^.

Choosing a companion during childbirth is a right and a practice strongly recommended for all women^(52)^, regardless of whether they are in prison. The presence of a companion throughout the entire labor period is essential, as it guarantees women physical and emotional support, well-being and safety, generating positive emotions and making this moment more humanized^(53)^.

Furthermore, it is essential to promote skin-to-skin contact between mother and baby early on. Women should be encouraged to do so immediately after birth. This practice consists of uninterrupted contact for one hour, with encouragement to breastfeed^(54)^, and is a crucial strategy for establishing the initial bond between mother and baby, offering several benefits to both^(55,56)^.

Considering another challenge reported by women, it is important to recognize that the lack of privacy^(27,28)^, combined with the incidence of verbal and psychological violence perpetrated by both prison officers and healthcare professionals^(24,26,28)^, is aggravated by societal discrimination^(6)^. This context expands the process of dehumanization faced by women already in vulnerable situations.

Given the multiple manifestations of obstetric violence, the need to implement specific measures to ensure dignified conditions and qualified care throughout the pregnancy-puerperal cycle of women deprived of liberty is evident. It is extremely important to adopt the guidelines recommended by the World Health Organization (WHO) for intrapartum care, aiming to provide a positive childbirth experience for women in labor^(52)^.

### Study Limitations

Despite the above, some limitations can be considered in this study, such as the methodological discrepancy between studies, cultural and legal diversity of the countries in which the research was conducted. The complexity of institutional dynamics and practices may have restricted the possibility of carrying out a more comprehensive analysis, which, in turn, may have reflected in the responses and experiences collected during the research.

### Advances for Health and Nursing

This review brings significant advances to the health and nursing field by identifying gaps in care during labor and delivery for women deprived of liberty. It offers essential insights to improve care practices, highlighting the urgency of humanized protocols.

## CONCLUSION

Scientific evidence points to the fragility of care practices for women deprived of their liberty during labor and childbirth, imposing significant challenges on women in labor, resulting in adverse experiences that compromise the quality of motherhood and violate women’s fundamental rights.

Assistance during childbirth is permeated by violence, including physical, verbal and psychological aspects, such as physical restraints, the use of handcuffs, lack of choice of companion, lack of skin-to-skin contact with the baby, lack of privacy and disrespectful attitudes on the part of prison officers and healthcare professionals, in addition to inadequate transportation conditions. Such practices not only disrespect basic rights, but also neglect women’s autonomy, contributing to traumatic and dehumanizing experiences during childbirth.

The review covers multivariate studies that identify discrepancies related to the environment, population and legislation. These elements represent challenges that restrict the uniformity of care during labor and childbirth for women deprived of liberty.

In this context, the need to implement public policies and guidelines to improve provision of healthcare to women deprived of liberty is highlighted. Moreover, the development of new research to fill the identified gaps is encouraged, aiming to produce scientific evidence that supports qualified management and promotes a positive childbirth experience for women deprived of liberty.
