# Supplementary material for: Labor and childbirth care for women deprived of liberty: a scoping review
Source: Rev Esc Enferm USP. 2024 Sep 13;58:e20240035. doi: 10.1590/1980-220X-REEUSP-2024-0035en (PMC11415216; doi:10.1590/1980-220X-REEUSP-2024-0035en)
Supplement: Translation

# Translation: Assistência ao trabalho de parto e parto de mulheres privadas de liberdade: revisão de escopo

**DOI:** 10.1590/1980-220X-REEUSP-2024-0035pt

**Authors:** Tatyanne Maria Pereira de Oliveira, Joel Araújo dos Santos, Priscila de Souza Aquino, Herla Maria Furtado Jorge

**Affiliations:** 1 Universidade Federal do Piauí, Departamento de Enfermagem, Teresina, PI, Brasil.; 2 Universidade Federal do Ceará, Departamento de Enfermagem, Fortaleza, CE, Brasil.

**Keywords:** Gestantes, Parturientes, Trabalho de Parto, Parto, Prisões

## Abstract

**Objetivo:**

Mapear e analisar as evidências científicas sobre a assistência prestada a mulheres privadas de liberdade durante o trabalho de parto e parto.

**Método:**

Revisão de escopo, desenvolvida em conformidade com o método JBI, cujas fontes de informação foram acessadas em bases de dados e na literatura cinzenta. A seleção foi realizada entre outubro e dezembro de 2023, a partir da leitura dos títulos, resumos e descritores, considerando os seguintes critérios de elegibilidade: artigos, dissertações e teses com diferentes desenhos metodológicos disponíveis na íntegra, sem delimitação de idioma e tempo. A análise foi conduzida por dois revisores independentes, utilizando-se a análise de conteúdo indutiva.

**Resultados:**

Foram incluídos 15 estudos. A partir da síntese dos resultados, emergiram duas categorias: Da cela para a sala de parto: assistência de mulheres privadas de liberdade; Experiências de mulheres privadas de liberdade durante o trabalho de parto e parto.

**Conclusão:**

Este estudo aponta a fragilidade das práticas assistenciais durante o trabalho de parto e parto, impondo desafios significativos e resultando em experiências adversas que comprometem a qualidade da maternidade e violam os direitos fundamentais das mulheres.

## INTRODUÇÃO

Assegurar o acesso à saúde é um direito fundamental que deve ser garantido a todas as pessoas^(1)^. No entanto, para grupos específicos da população, como as mulheres privadas de liberdade, existem obstáculos que resultam em uma precária assistência à saúde^(2)^. O encarceramento feminino intensifica os desafios relacionados às desigualdades sociais e à violência de gênero, especialmente no que se refere aos direitos sexuais e reprodutivos^(3)^.

No cenário internacional, apesar das políticas públicas que asseguram os direitos das mulheres privadas de liberdade, exemplificadas pelas Regras de Mandela de 1995 e as Regras de Bangok, promulgadas em 2010, ambas estabelecidas pela Organização das Nações Unidas (ONU)^(4,5)^, a implementação dessas diretrizes nas unidades prisionais continua a enfrentar diversos desafios. Essas adversidades incluem vulnerabilidades na atenção à saúde das mulheres durante a gestação até o puerpério, a ocorrência de violência obstétrica, experiências de abandono no momento do parto e inadequações no ambiente prisional^(6)^.

A literatura sinaliza para o aumento de gestantes em situação prisional, com uma taxa de crescimento global que passou de 7,2%, em 2006, para 8,8%, em 2014^(7)^. O ambiente prisional é caracterizado por condições desfavoráveis, marcado por espaços inadequados e insalubres. Essas condições não apenas representam um desafio para a saúde das gestantes, mas também têm o potencial de desencadear alterações biopsicossociais significativas^(8)^, estando associadas ao desenvolvimento de complicações materno-fetais^(9)^.

No contexto prisional, as gestantes são expostas à violência verbal, física e psicológica durante o pré-natal, o que acarreta implicações significativas durante o trabalho de parto e parto. Destacam-se práticas como a contenção física, seguidas pela negligência nos cuidados necessários durante o puerpério^(7)^. Essas práticas não estão alinhadas com os princípios de parto seguro^(10,11)^.

As dificuldades enfrentadas por gestantes nas prisões, marcadas por um acesso limitado aos cuidados de saúde e pela presença de violência psicológica e moral, ressaltam a necessidade premente de abordar essa lacuna. Esses desafios não apenas impactam as condições sociais e de saúde das mulheres privadas de liberdade, mas também têm repercussões nas vidas de seus filhos(as)^(12)^.

Nesse contexto, torna-se evidente a importância da atenção às parturientes em situação prisional, especialmente devido à carência de informações específicas. Destaca-se a ausência de estudos de revisão de escopo em andamento ou já concluídos sobre essa temática. Dessa forma, objetivou-se mapear e analisar as evidências científicas sobre a assistência prestada a mulheres privadas de liberdade durante o trabalho de parto e parto.

## MÉTODO

### Desenho do Estudo

Trata-se de revisão de escopo, elaborada conforme as recomendações do JBI, que tem como objetivo mapear as evidências científicas, principais conceitos e as lacunas sobre determinada temática^(13)^. Visando à qualidade e transparência do estudo, utilizaram-se as diretrizes contidas no *checklist Preferred Reporting Items for Systematic Reviews and Meta-Analyses extension for Scoping Reviews* (PRISMA-ScR)^(14)^. O protocolo de pesquisa foi registrado no *Open Science Framework* (OSF), com identificação do DOI: https://doi.org/10.17605/OSF.IO/MKAD5.

Destaca-se que a revisão de escopo representa um processo de síntese do conhecimento. Sua finalidade principal não reside na avaliação crítica da qualidade dessas evidências, mas sim na compreensão abrangente do conjunto de evidências encontradas^(14,15)^. Para tanto, foi realizada seguindo as cinco etapas: 1) elaboração da questão de pesquisa; 2) identificação de estudos relevantes; 3) seleção de estudos; 4) organização das informações; 5) síntese e apresentação dos resultados^(16)^.

### Identificação da Questão de Pesquisa

A questão de pesquisa foi elaborada a partir da estratégia mnemônica PCC, que corresponde a: P (População): gestantes ou parturientes; C (Conceito): assistência ao trabalho de parto e parto; e C (Contexto): privação de liberdade. Desta forma, elencou-se a seguinte questão: o que a literatura científica retrata sobre a assistência de mulheres privadas de liberdade durante o trabalho de parto e parto?

### Critérios de Seleção

Foram incluídos artigos, dissertações e teses com diferentes desenhos metodológicos relacionados à assistência ao trabalho de parto e parto de mulheres privadas de liberdade, disponíveis na íntegra, sem delimitação de idioma e tempo. Foram excluídos resumos de anais de evento, editoriais, carta resposta, reflexão teórica, trabalhos de conclusão de curso e aqueles que não responderam à questão de pesquisa.

### Coleta de Dados e Seleção dos Estudos

Para a realização das buscas, utilizaram-se os vocábulos controlados a partir dos Descritores em Ciência da Saúde (DeCS), *Medical Subject Headings* (MeSH), *List of Headings do CINAHL Information Systems*, *Embase Subject Headings* (EMTREE), identificando os descritores controlados e suas palavras-chave.

As buscas foram realizadas entre outubro e dezembro de 2023 nas seguintes bases de dados: *Medical Literature Analysis and Retrieval System Online* (MEDLINE) via *National Center for Biotechnology Information* (NCBI/PubMed); *Web of Science* (WoS); *Cumulative Index to Nursing and Allied Health Literature* (CINAHL); e *Excerpta Medica dataBASE* (EMBASE). Foram acessadas pelo Portal de Periódicos da Coordenação de Aperfeiçoamento de Pessoal de Nível Superior (CAPES), Literatura Latino-Americana e do Caribe em Ciências da Saúde (LILACS), Base de Dados em Enfermagem (BDENF), Índice Bibliográfico *Español en Ciencias de la Salud* (IBECS) via Biblioteca Virtual em Saúde (BVS). A literatura cinza também foi fonte de buscas por meio da Biblioteca Digital de Teses e Dissertações (BDTD) e *Google Scholar*.

O levantamento nas bases de dados ocorreu por meio da utilização dos descritores controlados e suas palavras-chave, combinados com os operadores booleanos “*OR*” e “*AND*” para compor a estratégia de busca. O Quadro 1 apresenta a expressão de busca de alta sensibilidade realizada na base MEDLINE/PubMed, que foi adaptada para as demais bases de dados selecionadas de acordo com suas especificidades e podem ser verificadas no protocolo de revisão de escopo: https://doi.org/10.17605/OSF.IO/MKAD5.

**Quadro 1:** Expressão de busca de alta sensibilidade realizada na base MEDLINE/PubMed – Teresina, PI, Brasil, 2023.

MEDLINE/PubMed
*((“Pregnant Women”[Mesh] OR (Pregnant Woman) OR (Woman, Pregnant) OR (Women, Pregnant) OR “Pregnancy”[Mesh] OR (Pregnancies) OR (Gestation)) AND (“Labor, Obstetric”[Mesh] OR (Obstetric Labor) OR “Delivery, Obstetric”[Mesh] OR (Deliveries, Obstetric) OR (Obstetric Deliveries) OR (Obstetric Delivery) OR “Parturition”[Mesh] OR (Parturitions) OR (Birth) OR (Births) OR (Childbirth) OR (Childbirths))) AND (“Prisons”[Mesh] OR (Prison) OR (Penitentiaries) OR (Penitentiary) OR “Prisoners”[Mesh] OR (Prisoner) OR (Hostages) OR (Hostage) OR “Correctional Facilities”[Mesh] OR (Correctional Facility) OR (Facilities, Correctional) OR (Facility, Correctional) OR (Penal Institutions) OR (Institution, Penal) OR (Institutions, Penal) OR (Penal Institution) OR (Correctional Institutions) OR (Correctional Institution) OR (Institution, Correctional) OR (Institutions, Correctional))*

Após as buscas nas bases de dados, os resultados foram importados para o programa de gerenciamento de referências, o *EndNote* ^®^ *Web*, para identificação de duplicatas. Em seguida, os resultados foram importados para o aplicativo Rayyan^®^ do *Qatar Computing Research Institute* (QCRI)^(17)^ para análise, seleção e exclusão dos estudos. Ressalta-se que as etapas foram conduzidas por dois revisores independentes. Os casos de discordância foram resolvidos com a ajuda de um terceiro revisor, antes de prosseguir para a leitura completa e inclusão dos estudos na revisão.

A primeira seleção foi realizada por meio da leitura de títulos e resumos para análise da concordância dos autores. Foi realizado o cálculo do coeficiente Kappa de Cohen^(18)^, que apresentou valor de 0,85 (concordância forte). Posteriormente, os artigos selecionados foram lidos na íntegra e avaliados conforme os critérios de inclusão e exclusão.

Os dados foram extraídos, de acordo com o instrumento adaptado do manual do JBI^(13)^, em quadros sinópticos no *software Microsoft Excel* ^®^ contendo as informações sobre autoria, periódico, país de origem, ano de publicação, título do estudo, objetivo, desenho de pesquisa, número da amostra e assistência acerca do trabalho de parto e parto.

### Análise dos Dados

Os dados foram submetidos à análise de conteúdo indutiva^(19)^. Esse processo foi estruturado seguindo as três etapas propostas: preparação dos dados; organização; e relatório. Na etapa inicial de preparação, os dados foram organizados em quadros sinópticos, conforme as informações previamente estabelecidas. Em seguida, durante a etapa de organização, os principais resultados foram identificados e submetidos à codificação aberta para posterior categorização. Por fim, na etapa de elaboração do relatório, que corresponde à redação final do material, os resultados foram apresentados de forma descritiva por meio de quadros e textos, e foram agrupados em categorias temáticas comuns, proporcionando uma síntese clara e concisa dos achados. Além disso, utilizamos o *software Interface de R pour les Analyses Multidimensionnelles de Textes et de Questionnaires* (IRaMuTeQ)^(20)^ apenas para a criação da nuvem de palavras baseada nos resultados obtidos sobre a assistência ao trabalho de parto e parto das publicações selecionadas.

Por se tratar de estudo de revisão de escopo, não houve a necessidade de avaliação por Comitê de Ética e Pesquisa (CEP).

## RESULTADOS

Foram identificadas 995 publicações a partir da estratégia de busca, sendo 195 na MEDLINE/PubMed, 135 na WoS, 150 na CINAHL, 123 na EMBASE, 51 na LILACS, 32 na BDENF, 12 na IBECS, 102 na BDTD e 195 no *Google Scholar*. Foram excluídos 299 registros por duplicidade. Mantiveram-se 696 publicações elegíveis para a etapa de análise de título e resumo; dessas, houve a exclusão de 612 estudos. Logo, 84 foram selecionados para leitura do texto completo e análise para inclusão da revisão, dos quais 69 foram excluídos. Assim, 15 estudos foram selecionados para a síntese final. As etapas de seleção foram realizadas de acordo com o fluxograma PRISMA-ScR, conforme descrito na Figura 1.

**Figura 1:** Fluxograma do processo de seleção dos estudos conforme o *Preferred Reporting Items for Systematic Reviews and Meta-Analyses extension for Scoping Reviews* (PRISMA-ScR) – Teresina, PI, Brasil, 2023.

O Quadro 2 mostra os 15 estudos que compuseram a amostra final da pesquisa. A partir da análise dos mesmos, emergiram as categorias: “Da cela para a sala de parto: assistência de mulheres privadas de liberdade”^(21-24,27,29,30,33)^; e “Experiências de mulheres privadas de liberdade durante o trabalho de parto e parto”^(21-28,30-35)^.

**Quadro 2:** Caracterização das pesquisas que compõem a amostra do estudo de acordo com os dados de autoria, periódico, país e ano, título do artigo, objetivos, desenho de pesquisa, número da amostra e assistência ao trabalho de parto e parto – Teresina, PI, Brasil, 2023 (n = 15).

Autoria/periódico/país e ano	Título	Objetivo	Desenho de pesquisa/nº amostra	Assistência acerca do trabalho de parto e parto
Abbott et al.^(21)^. *Birth*, Reino Unido/2023	*Experiences of midwifery care in English prisons*	Examinar as experiências e percepções de cuidados obstétricos de mulheres grávidas detidas e funcionários de custódia em prisões inglesas.	Estudo qualitativo (etnografia)/28 mulheres	– Não foi dada a escolha de parceiro para o parto e nascimento; – Não havia escolha do prestador de cuidados obstétricos ou do local de nascimento; – Tanto as mulheres quanto o pessoal demonstraram falta de consciência dos direitos que uma mulher grávida deveria receber.
Kramer et al.^(22)^. *Matern Child Health J*. Estados Unidos/2023	*Shackling and pregnancy care policies in US prisons and jails*	Avaliar políticas e práticas de gravidez em prisões e cadeias com ênfase no uso de restrições e conformidade com legislação anti-algemamento.	Estudo qualitativo/28 unidades	– Restrições foram usadas durante a gravidez e pós-parto, inclusive durante o transporte; – Isolamento significativo para a maioria das mulheres que dão à luz sob custódia; – Não exigiam que o oficial presente durante o parto fosse do sexo feminino.
Cavanagh et al.^(23)^. *Soc Sci Med*. Canadá/2022	*Lived experiences of pregnancy and prison through a reproductive justice lens: A qualitative meta-synthesis*	Compreender melhor como as pessoas encarceradas vivenciam a gravidez e como suas experiências são representadas na pesquisa.	Revisão sistemática (metassi´ntese)/31 artigos	– O trabalho de parto e o nascimento foram traumatizantes para muitas mulheres, exacerbados pela intersecc¸a~o de poli´ticas carcera´rias e pra´ticas cli´nicas que desconsideravam a sua autonomia corporal e o seu papel como ma~e; – Uma participante descreveu ter sido agendada e transportada para o hospital para dar a` luz sem saber o que iria acontecer.
Dalenogare et al.^(24)^. Ciên Saúde Colet. Brasil/2022	Pertencimentos sociais e vulnerabilidades em experie^ncias de parto e gestac¸a~o na prisa~o	Compreender as experiências de gestação e parto de mulheres em situação prisional.	Estudo qualitativo/sete mulheres	– O trabalho de parto foi percebido como doloroso e angustiante, pelo estado degradante da assistência ofertada desde a remoção da unidade prisional até a instituição de saúde; – Na penitenciária, o trabalho de parto tem sua evolução acompanhada pelas profissionais da Unidade Básica de Saúde Prisional; – Durante o período noturno ou em fins de semana, a decisão de encaminhamento da mulher à instituição de saúde e´ realizada pelos agentes de segurança; – Ausência de acompanhantes da escolha da mulher e, muitas vezes, sem poder avisa´-los sobre a situação, elas são acompanhadas pelas agentes de segurança; – Uso de algemas no trajeto da penitenciária até a instituição de saúde; – O tratamento oferecido pela equipe da instituição de saúde e´ percebido de diferentes formas pelas mulheres. Algumas elogiaram e outras relataram ser tratadas com indiferença, negligência e violência; – Desatenção às boas práticas relacionadas ao parto, com o uso de medidas intervencionistas para sua celeridade.
Kirubarajan et al.^(25)^. BJOG-Int J *Obstet Gy*. Canadá/2022	*Pregnancy and childbirth during incarceration: A qualitative systematic review of lived experiences*	Caracterizar as experiências dos pacientes em relação à gravidez e ao parto durante o encarceramento por meio de síntese qualitativa.	Revisão sistemática (metassi´ntese)/24 artigos	– Uso de restrições durante a gravidez e o parto; – Falta de apoio emocional; – O trauma da separação do recém-nascido após o nascimento.
Fortunato et al.^(26)^. REAS. Brasil/2022	Percepção das mulheres privadas de liberdade sobre a assistência à saúde recebida no pré-natal, parto e puerpério: revisão integrativa	Descrever, do ponto de vista das mulheres privadas de liberdade, como ocorre a assistência à saúde durante a gestação, parto e puerpério.	Revisão integrativa/15 artigos	– Demora no encaminhamento à maternidade; – Familiares não são informados do início do trabalho de parto e, quando avisados, não conseguem chegar a tempo para acompanhar a mulher; – A escolta policial não efetiva o contato com a família, e são escassas as visitas que essas mulheres recebem ainda na maternidade; – Mulheres destacaram a violência, principalmente na forma verbal e psicológica, além dos procedimentos invasivos, agressão física e negligência, falta humanização e orientação durante o atendimento; – Há relatos do uso de algemas durante o parto e a internação.
Suarez^(27)^. J *Correct Health Care*. Estados Unidos/2021	*“I Wish I Could Hold Your Hand”: Inconsistent Interactions Between Pregnant Women and Prison Officers*	Explorar as interações das mulheres com os agentes penitenciários durante a gravidez, trabalho de parto e parto.	Estudo qualitativo/18 mulheres	– Agentes rudes na unidade durante os transportes e até mesmo na sala de parto; – Falta geral de privacidade e de reconhecimento de que estavam a dar a` luz; – Os policiais às vezes eram intrometidos ou muito faladores ou ignoravam a parturiente que muitas vezes sentia dor.
Abbott et al.^(28)^. *Weston. Sociol Health Illn*. Reino Unido/2020	*Pregnancy and childbirth in English prisons: institutional ignominy and the pains of imprisonment*	Explorar as experiências de mulheres grávidas em prisões por meio de entrevistas qualitativas com uma amostra de mulheres detidas, outra amostra de funcionários prisionais e observações de campo.	Estudo qualitativo (etnografia)/28 mulheres	– A perda de privacidade agravou a perda de dignidade e decência; – O ambiente era considerado tão hostil ao trabalho de parto espontâneo que parecia inseguro entrar em trabalho de parto na prisão.
Johnston^(29)^. *Criminol Crim Justice*. Reino Unido/2019	*Imprisoned mothers in Victorian England, 1853–1900: Motherhood, identity and the convict prison*	Explorar as experiências de mães encarceradas no sistema prisional de condenados da era vitoriana.	Estudo qualitativo (história de vida)/288 mulheres	– Grávidas quando foram detidas ou condenadas deram a` luz nas prisões locais.
Matos et al.^(30)^. Interface. Brasil/2019	Filhos do cárcere:representac¸o~es sociais de mulheres sobre parir na prisa~o	Compreender as representações sociais de gestantes e puérperas encarceradas sobre o parir na prisão.	Estudo qualitativo/19 mulheres	– Presença apenas dos agentes penitenciários quando o momento de parir e´ chegado e são direcionadas ao hospital; – Percebem o julgamento impetrado, denotando o preconceito de outras parturientes, acompanhantes e profissionais de saúde para com elas.
Leal et al.^(31)^. Ciên Saúde Colet. Brasil/2016	Nascimento na prisão: gravidez e nascimento atrás das grades no Brasil	Traçar o perfil da população feminina encarcerada que vive com seus filhos em unidades prisionais femininas das capitais e regiões metropolitanas do Brasil, bem como as condições e as práticas relacionadas à atenção à gestação e ao parto durante o encarceramento.	Estudo quantitativo/241 mães	– A presença de acompanhantes da escolha da mulher durante a internação para o parto foi de 3%; – As puérperas relataram ter sofrido maus-tratos ou violência durante a estadia nas maternidades pelos profissionais de saúde (16%) e pelos guardas ou agentes penitenciários (14%); – O uso de algemas em algum momento da internação para o parto foi referido por 36% das gestantes, sendo que 8% relataram ter ficado algemadas mesmo durante o parto; – Apenas 10% e 11% das mulheres referiram ter sido respeitadas quanto à sua intimidade pelos profissionais de saúde e pelos guardas/agentes penitenciários, respectivamente. Esse percentual foi um pouco maior quando o tema foi o trato dos profissionais de saúde com elas (18%).
Spinola^(32)^. Faculdade de Medicina da USP. Brasil/2016	A experiência da maternidade no cárcere: cotidiano e trajetórias de vida	Conhecer e compreender a experiência da maternidade no cárcere a partir do cotidiano e da trajetória de vida de mulheres egressas do sistema penitenciário.	Estudo qualitativo (hermenêutica)/duas mulheres	– Cumprimento de todos os procedimentos burocráticos (assinaturas, revistas) para depois ser encaminhada para o hospital; – A condição de ser levada para o hospital se deu apenas quando a criança estava prestes a nascer; – Uso de algemas nos pés e mãos durante o trabalho de parto e parto; – Sensação de dor e sem comunicação adequada; – Descrição do parto com dificuldades.
Ferszt e Clarke^(33)^. *J Health Care Poor Underserved*. Estados Unidos/2012	*Health care of pregnant women in U.S. state prisons*	Examinar as práticas de cuidados de saúde de mulheres grávidas em prisões estaduais.	Estudo misto/32 prisões	– Uso de correntes/cintos abdominais, grilho~es nas pernas e algemas ao transportar mulheres para um hospital ou clínica; – Contenção durante o trabalho de parto e mesmo durante o nascimento do bebe^; – Contenção durante o período de recuperação imediata e nos quartos do hospital.
Rosinski et al.^(34)^. Ciênc., Cuid. Saúde. Brasil/2006	Nascimento atra´s das grades: uma pra´tica de cuidado direcionada a gestantes, pue´rperas e rece´m-nascidos em privac¸a~o de liberdade	Desenvolver uma prática de cuidado direcionada a gestantes, puérperas e recém-nascidos em privação de liberdade, orientada pela Teoria Geral de Enfermagem de Orem.	Estudo qualitativo/12 mulheres	– Uso de algemas durante o trabalho de parto e parto que impossibilitou segurar o bebê; – Ausência de contato com a criança.
Amnesty International^(35)^. *Birth*. Estados Unidos/2000	*Pregnant and imprisoned in the United States*	Descrever violações dos direitos humanos de mulheres grávidas e mães encarceradas em prisões e cadeias nos Estados Unidos.	Estudo qualitativo/-	– Grávidas eram contidas quando eram transportadas para o hospital e mantidas sob restrições enquanto estavam no hospital, mesmo durante o trabalho de parto, a menos que um médico solicitasse a remoção e um oficial correcional aprovasse; – Mulheres eram algemadas mesmo na presença de um agente penitenciário ou, em alguns casos, as algemas eram removidas até 30 minutos antes do parto; – Ausência de permissão para movimentação durante o trabalho de parto; – Algumas mulheres relataram que, após o nascimento, permaneceram por um tempo com o bebê, mas, logo em seguida, os policiais recolocaram as algemas para fazer a remoção da sala de parto.

Quanto ao tipo de estudo, selecionaram-se 14 artigos e uma dissertação. O ano de publicação variou entre 2000 e 2023, com predomínio de publicações no ano de 2022 (n = 4). No que se refere ao local de publicação, o Brasil foi o país com maior número de estudos (n = 6), seguido dos Estados Unidos (n = 4), Reino Unido (n = 3) e Canadá (n = 2). Quanto ao idioma, a maioria dos estudos foram publicados em inglês (n = 9) e em português (n = 6). Em relação às características metodológicas das publicações incluídas neste estudo, destacaram-se pesquisas qualitativas (n = 10), revisões (n = 3), estudos mistos (n = 1) e quantitativos (n = 1).

### Da Cela Para a Sala de Parto: Assistência de Mulheres Privadas de Liberdade

O parto representa uma ruptura súbita da ligação estabelecida no pré-natal, sendo traumatizante para muitas mulheres devido às políticas prisionais e práticas clínicas que desconsideram sua autonomia corporal e seu papel como mãe^(23)^. A assistência ao trabalho de parto e parto inicia-se ainda no ambiente prisional, no qual os profissionais de saúde da unidade prisional monitoram o trabalho de parto e parto, orientando o momento adequado para encaminhar a paciente aos hospitais de referência, no entanto, no período noturno ou nos fins de semana, essa decisão é tomada pelos agentes de segurança^(24,30)^.

Nesse contexto, a assistência é caracterizada pela falta de privacidade, desrespeito ao trabalho de parto e negligência ou desconsideração da dor relatada, contribuindo para a demora no atendimento, aumentando a sensação de abandono e, consequentemente, levando algumas mulheres a dar à luz sozinhas, sem assistência^(27)^, e muitas vezes as mulheres grávidas pariam na própria prisão^(29)^.

Atualmente, é recomendada a transferência da parturiente para assistência hospitalar. No entanto, observa-se que o transporte muitas vezes é realizado de maneira inadequada, incluindo práticas como a restrição de movimentos com o uso de contenção (algemas, correntes, grilhões) nas pernas e o uso de correntes/cintos abdominais, apesar de entendimento internacional e proibições legais contrárias^(22,24,33)^.

A inclusão de um agente de segurança do sexo masculino durante o transporte foi mencionada como uma redução da autonomia e uma ausência de escolha, provocando desconforto, insegurança e ansiedade, constituindo uma fonte de estresse^(21)^. O parto é reconhecido como um momento de angústia, dor e solidão, não apenas devido aos aspectos fisiológicos, mas também devido à degradação na qualidade da assistência oferecida, apesar de alguns relatos de cuidados eficazes^(24)^.

### Experiências de Mulheres Privadas de Liberdade Durante o Trabalho de Parto e Parto

Mulheres testemunham a negligência em relação às boas práticas relacionadas ao parto, que é agravada pelo uso de contenções, como algemas e correntes, na cama durante o processo de parturição. Relatos mencionam que pernas, mãos e até mesmo a coluna (abdome) são locais de acorrentamento^(22,24-26,31,32,34,35)^. É destacado que as contenções são removidas somente mediante solicitação médica^(22,33,35)^.

Outra questão apontada é a ausência de um acompanhante escolhido livremente ou a presença de um agente de segurança do sexo masculino, que indica um isolamento significativo, podendo resultar em experiências traumáticas, humilhantes e desencadear traumas sexuais^(22,26-28)^. Ademais, o contato precoce entre a mãe e bebê é mínimo ou inexistente^(25,34)^, o que pode acarretar impactos negativos no desenvolvimento da criança e, também, para as mulheres. Além disso, após o parto, algumas mulheres foram novamente algemadas, sendo a separação do bebê descrita como um evento traumático e devastador para a mãe^(25)^.

Tanto as mulheres quanto outros envolvidos demonstram falta de consciência em relação aos direitos que deveriam ser garantidos a uma mulher grávida^(21)^. Esses aspectos contribuem para que as mulheres reconheçam sua falta de preparo durante o parto, com um desconhecimento geral do processo^(30)^.

Na Figura 2, ilustramos as principais palavras identificadas na análise das publicações acerca da assistência durante o trabalho de parto e parto de mulheres privadas de liberdade.

**Figura 2:** Nuvem de palavras dos estudos sobre a assistência durante o trabalho de parto e parto – Teresina, PI, Brasil, 2023.

## DISCUSSÃO

A assistência de mulheres privadas de liberdade é marcada por desafios significativos, conforme apontam os estudos^(21–24)^. A situação de privação muitas vezes é caracterizada por restrições rigorosas, isolamento social e consequências para transgressões legais. Implicitamente, surge a imagem de privação de direitos, maus-tratos e dor. Essa percepção se estende ao cuidado de mulheres grávidas privadas de liberdade, desde o pré-natal até o pós-parto.

Para uma análise abrangente da situação, é crucial examinar a saúde das mulheres em ambiente prisional de forma geral. Estudo realizado no Ceará, Brasil, em 2018^(2)^, com mulheres privadas de liberdade revelou que a qualidade da assistência à saúde nas instalações prisionais investigadas é insatisfatória. Além da negligência em relação às necessidades específicas do sexo feminino, há uma carência significativa de cuidados básicos, como alimentação adequada, vestuário, produtos de higiene e medicamentos.

A população carcerária feminina requer uma abordagem específica que leve em consideração suas particularidades sociais e culturais, pois esses são fatores cruciais no planejamento de intervenções apropriadas^(2)^. Ao concentrar-se apenas na dimensão da maternidade como parte da identidade feminina, as instituições prisionais reforçam os estereótipos de gênero presentes na sociedade em geral, limitando a visão das mulheres apenas ao papel de mães^(36)^.

Em relação ao parto, considerado um período de angústia, dor e solidão, representa uma ruptura súbita da ligação estabelecida durante o pré-natal. Muitas vezes, é descrito como uma experiência traumática^(23)^ para várias mulheres encarceradas, não apenas devido a fatores fisiológicos, mas também pela falta de qualidade na assistência oferecida^(37)^.

A literatura evidencia as precárias condições sociais das mães que pariram dentro do contexto de privação de liberdade. Entre várias questões, o acesso limitado aos cuidados de saúde, o emprego de algemas durante o trabalho de parto e parto, juntamente com relatos de violência e avaliações negativas do atendimento, indicam que os serviços de saúde não têm desempenhado efetivamente seu papel como uma barreira protetora e garantidora dos direitos desse segmento da população^(6,38,39)^.

Estudos conduzidos no Peru^(40)^, Reino Unido^(41)^ e nos Estados Unidos^(42)^ apontam que, após o diagnóstico de gravidez, mulheres privadas de liberdade são transferidas para unidades maternas com maior adequação de suas necessidades, no entanto nem todas têm acesso garantido às vagas nessas unidades. No contexto brasileiro, em grande parte dos estados, mulheres grávidas também são transferidas para unidades prisionais específicas para mães com filhos^(6,9)^. Quanto ao momento do parto, essas mulheres são encaminhadas a hospitais públicos^(37,43–45)^ e, após o nascimento, retornam à mesma unidade prisional, onde muitas vezes permanecem com seus filhos. Posteriormente, as crianças são geralmente entregues aos familiares maternos ou paternos e, na ausência destes, encaminhadas para abrigos, enquanto a mãe retorna à prisão de origem^(31)^.

Ao lado de instrumentos normativos internacionais, como as Regras de Bangkok^(5)^, e das políticas nacionais sobre direitos reprodutivos nas prisões que, embora importantes, são pouco respeitadas no Brasil, a Portaria Interministerial, de 16 de janeiro de 2014^(46)^, representa uma mudança significativa na abordagem das questões relacionadas ao encarceramento feminino no país^(31)^, uma vez que o aumento contínuo no número de mulheres privadas de liberdade e o reconhecimento da complexidade desse fenômeno destacam a urgência de uma reflexão mais ampla sobre questões relacionadas à gravidez e à maternidade nesse contexto^(36)^.

Na análise da segunda categoria, relacionada às experiências das mulheres privadas de liberdade durante o trabalho de parto e parto, é evidente que elas frequentemente descrevem suas vivências maternas como uma sequência de desafios enfrentados ao longo de todo o período gestacional^(23,31)^. Os resultados revelam uma série de dificuldades que não apenas comprometem a qualidade da maternidade, mas também violam os direitos fundamentais dessas mulheres, manifestando-se por meio de violência física, verbal e psicológica.

Entre as principais violências, a prática de impor restrições físicas é preocupante, pois transcende os limites da segurança, comprometendo o ajuste fisiológico do corpo devido à limitação de movimentos, intensificando a dor e dificultando a dinâmica do parto. Considerando a diversidade cultural, legal e institucional entre diferentes países, no contexto brasileiro onde se concentrou o maior número de estudos, a Lei nº 13.434/2017 proíbe o uso de algemas durante o trabalho de parto, parto e puerpério imediato^(47)^. No Canadá e no Reino Unido, as diretrizes jurisdicionais abordam o uso de algemas e/ou contenções físicas, preconizando a sua não utilização, mas permitem seu emprego quando julgado necessário^(48–50)^. Nos Estados Unidos, a lei não é uniforme entre os estados, o que resulta em uma variedade de legislações que mencionam a proibição e utilização em algum momento da gestação e parto^(48)^.

Nesse sentido, embora a ONU proíba o uso das algemas durante o trabalho de parto e parto^(5)^, a diversidade legal entre os países contribui para disparidades na prestação de assistência à saúde para gestantes e parturientes em situação de privação de liberdade.

Além disso, mulheres em situação prisional têm relatado outras formas de violência obstétrica, como a falta de um acompanhante de escolha durante o parto^(20,23,25,30)^. Em contrapartida, no Reino Unido, algumas unidades prisionais adotam uma abordagem diferente, permitindo que as mulheres solicitem a presença de um agente penitenciário de sua confiança durante o trabalho de parto^(51)^. Em situações especiais, como as Unidades Materno-Bebê, quando as mulheres conseguem ingressar, têm acesso a acompanhantes de parto vinculados a instituições de caridade, proporcionando um suporte essencial durante esse momento tão significativo^(41)^.

A escolha do acompanhante durante o parto é um direito e uma prática fortemente recomendada a todas as mulheres^(52)^, independentemente de estarem em situação prisional. A presença do acompanhante durante todo o período de parturição é essencial, pois garante às mulheres suporte físico, emocional, bem-estar e segurança, gerando emoções positivas e tornando esse momento mais humanizado^(53)^.

Além disso, é fundamental promover precocemente o contato pele a pele entre mãe e bebê. Deve-se incentivar as mulheres logo após o nascimento. Essa prática consiste no contato por uma hora ininterrupta, com estímulo à amamentação^(54)^, e é uma estratégia crucial para estabelecer o vínculo inicial entre mãe e bebê, oferecendo diversos benefícios para ambos^(55,56)^.

Considerando um outro desafio relatado pelas mulheres, é importante reconhecer que a ausência de privacidade^(27,28)^, aliada à incidência de violência verbal e psicológica perpetrada tanto por agentes penitenciários quanto por profissionais de saúde^(24,26,28)^, é agravada pela discriminação da sociedade^(6)^. Esse contexto amplia o processo de desumanização enfrentado por mulheres já em situação de vulnerabilidade.

Diante das múltiplas manifestações de violência obstétrica, evidencia-se a necessidade de implementar medidas específicas para garantir condições dignas e assistência qualificada durante todo o ciclo gravídico-puerperal de mulheres privadas de liberdade. É de suma relevância adotar as diretrizes preconizadas pela Organização Mundial da Saúde (OMS) para os cuidados intraparto, visando proporcionar uma experiência de parto positiva para as parturientes^(52)^.

### Limitações do Estudo

A despeito do exposto, algumas limitações podem ser consideradas neste estudo, como a discrepância metodológica entre os estudos, diversidade cultural e legal dos países nos quais as pesquisas foram conduzidas. A complexidade das dinâmicas e práticas institucionais pode ter restringido a possibilidade de realizar uma análise mais abrangente, o que, por sua vez, pode ter refletido nas respostas e experiências coletadas durante a pesquisa.

### Avanços Para a Área da Saúde e Enfermagem

Esta revisão traz avanços significativos para a área da saúde e enfermagem, ao identificar as lacunas na assistência durante o trabalho de parto e parto de mulheres privadas de liberdade. Oferece *insights* essenciais para melhorar as práticas assistenciais, evidenciando a urgência de protocolos humanizados.

## CONCLUSÃO

As evidências científicas apontam a fragilidade das práticas assistenciais para as mulheres privadas de liberdade durante o trabalho de parto e parto, impondo desafios significativos às parturientes, resultando em experiências adversas que comprometem a qualidade da maternidade e violam os direitos fundamentais das mulheres.

A assistência ao momento de parturição é permeada por violência, incluindo aspectos físicos, verbais e psicológicos, como restrições físicas, com o uso de algemas, a falta de escolha de acompanhante, a ausência de contato pele a pele com o bebê, a falta de privacidade e atitudes desrespeitosas por parte de agentes penitenciários e profissionais de saúde, além de condições inadequadas de transporte. Tais práticas não apenas desrespeitam os direitos básicos, mas também negligenciam a autonomia das mulheres, contribuindo para experiências traumáticas e desumanizadoras durante o parto.

A revisão abrange estudos multivariados que identificam discrepâncias relacionadas ao ambiente, à população e à legislação. Esses elementos representam desafios que restringem a uniformidade dos cuidados durante o trabalho de parto e parto para mulheres privadas de liberdade.

Neste contexto, ressalta-se a necessidade da implementação de políticas públicas e diretrizes para aprimorar a prestação de cuidados de saúde a mulheres em privação de liberdade. Além disso, incentiva-se o desenvolvimento de novas pesquisas para preencher as lacunas identificadas, visando à produção de evidências científicas que embasem o manejo qualificado e promovam uma experiência de parto positiva para as mulheres privadas de liberdade.
